# Haemolytic Anaemia; Beyond Sickle Cell Anaemia: A Case Series of Patients With Paroxysmal Nocturnal Haemoglobinuria in Kenya

**DOI:** 10.1155/crh/1756509

**Published:** 2026-07-29

**Authors:** Matilda Ong’ondi, Otom Lavender, Muraya Gathinji, Zaheer Bagha

**Affiliations:** ^1^ Hemato-Oncology Unit, Kenyatta National Hospital, Nairobi, Kenya, knh.or.ke; ^2^ Department of Clinical Medicine and Therapeutics, University of Nairobi, Nairobi, Kenya, uonbi.ac.ke; ^3^ Fellow ECSACOP, Coast General Teaching and Referral Hospital, Mombasa, Kenya; ^4^ Coast General Teaching and Referral Hospital, Mombasa, Kenya

**Keywords:** case series, haemolytic anaemia, paroxysmal nocturnal haemoglobinuria (PNH)

## Abstract

Paroxysmal nocturnal haemoglobinuria (PNH) is a rare, acquired clonal haematopoietic stem cell disorder characterised by intravascular haemolysis, thrombosis and bone marrow failure. It results from an acquired somatic mutation in the X‐linked phosphatidylinositol glycan Class A (PIGA) gene, leading to deficiency of glycosylphosphatidylinositol‐anchored complement regulatory proteins, particularly CD55 and CD59, and uncontrolled complement‐mediated haemolysis. Although sickle cell disease is the most prevalent haemolytic anaemia in Kenya, other aetiologies need to be considered. We describe a case series of seven patients with PNH confirmed by fluorescent aerolysin reagent (FLAER) flow cytometry. Patients were young, aged 17–38 years (median age of 23 years), with a predominance of males. All presented with recurrent symptomatic anaemia requiring multiple transfusions. One patient had pancytopenia due to very severe aplastic anaemia, while another presented with mesenteric vein thrombosis complicated by bowel ischaemia. All patients had large PNH clones on flow cytometry. Management was largely supportive due to a lack of access to complement inhibitor. These included blood transfusion, folate supplementation, cautious iron replacement and anticoagulation where indicated. One patient underwent successful matched sibling allogeneic stem cell transplant. This series highlights the presence of PNH in Kenya, diagnostic delay and the need for improved access to definitive testing and therapy in resource‐limited environments.

## 1. Introduction

Paroxysmal nocturnal haemoglobinuria (PNH) is a rare acquired X‐linked clonal haematopoietic stem cell disorder characterised by haemolytic anaemia, thrombosis and bone marrow failure [1–3]. PNH results from an acquired somatic mutation in the phosphatidylinositol glycan Class A (PIGA) gene located on the X chromosome. PIGA is X‐linked; therefore, a single acquired somatic mutation in a haematopoietic stem cell is sufficient to produce the PNH phenotype. This applies to males, who have a single X chromosome, and to females, in whom lyonization results in only one active X chromosome [4].

This mutation leads to defective synthesis of glycosylphosphatidylinositol (GPI) anchors, which are required for the attachment of complement regulatory proteins, most notably CD55 and CD59, to the cell surface. The absence of these protective proteins renders erythrocytes susceptible to uncontrolled complement‐mediated intravascular haemolysis and contributes to activation of platelets, monocytes and granulocytes [1, 4]. In patients treated with terminal complement inhibitors, haemolysis may persist in the extravascular compartment due to C3 fragment (C3d) opsonization of GPI‐deficient erythrocytes, which are subsequently cleared by the reticuloendothelial system [1].

PNH is a rare disorder with variable incidence and prevalence across populations. A population‐based cohort study from the United Kingdom reported an incidence of 0.35 cases per 100,000 person‐years and a prevalence of 3.81 per 100,000, while a separate retrospective cohort study estimated prevalence at 12‐13 per million and an incidence of 5.7 per million during the study period [5, 6]. PNH is exceedingly rare in Africa, with the literature limited to isolated case reports and small case series, collectively accounting for fewer than 50 reported cases to date [7–15].

We present a case series of seven patients with PNH confirmed by fluorescent aerolysin reagent (FLAER) flow cytometry, with the aim of increasing awareness of this rare but serious condition and highlighting the importance of considering PNH in patients with unexplained haemolytic anaemia once more common causes have been excluded.

## 2. Clinical Cases

The patients were predominantly young, with an age range of 17–38 years and a median age of 23 years (Table 1). There was a male predominance, with only one female patient in the cohort.

**TABLE 1:** Demographic and clinical characteristics.

	Patient 1	Patient 2	Patient 3	Patient 4	Patient 5	Patient 6	Patient 7
Age (years)	23	28	38	17	26	20	23
Sex	Male	Male	Male	Male	Male	Female	Male
Duration of symptoms prior to diagnosis	6 years	7 years	10 years	2 months	2 years	2 years	8 years
Presenting symptoms	Anaemia, dark urine, jaundice	Anaemia	Anaemia, dark urine, jaundice, abdominal pain	Anaemia, epistaxis	Anaemia, dark urine, jaundice	Anaemia, dark urine, jaundice, abdominal pain	Anaemia, dark urine
Hepatosplenomegaly (yes/no)	No	No	No	No	No	No	No
History of multiple transfusions (yes/no)	Yes	Yes	Yes	Yes	Yes	Yes	Yes
Thrombotic events	No	No	Yes	No	No	No	No
Site of thrombosis	N/A	N/A	Superior mesenteric vein	N/A	N/A	N/A	N/A
Treatment received	Supportive	Supportive	Supportive	EPAG, cyclosporin, HSCT	Supportive	Supportive	EPAG, cyclosporin
Current status	Transfusion‐dependent.	Transfusion‐dependent	Transfusion‐dependent.	Post‐HSCT	Transfusion‐dependent	Transfusion‐dependent	Transfusion‐dependent

*Note:* EPAG, Eltrombopag.

Abbreviations: HSCT, haematopoietic stem cell transplant; NA, not applicable.

All patients presented with a history of recurrent symptomatic anaemia, manifesting as easy fatigability, palpitations, exertional dyspnoea and headache. They had all required multiple blood transfusions at various time points prior to diagnosis. With the exception of Patient 4 who had a symptom duration of 2 months, the remaining patients had a prolonged disease course with symptoms and transfusion dependence for a mean duration of 5.02 years (median 6.0 years; range 2 months to 10 years) from symptom onset to diagnosis of PNH.

Clinical features suggestive of haemolysis were common. Jaundice was reported in four patients, while five patients described episodes of dark‐coloured urine, predominantly occurring at night. Abdominal pain was reported in two patients (Patients 3 and 6). On physical examination, pallor was universally present, and jaundice was noted in some patients; however, none had lymphadenopathy or hepatosplenomegaly. One patient (Patient 3) presented with an acute abdomen and was found intraoperatively to have bowel ischaemia secondary to superior mesenteric vein thrombosis, requiring bowel resection and anastomosis.

Baseline investigations performed as part of the anaemia workup are summarised in Table 2.

**TABLE 2:** Laboratory findings at diagnosis.

Investigation	Patient 1	Patient 2	Patient 3	Patient 4	Patient 5	Patient 6	Patient 7
Haemoglobin (g/dL)	6.7	2.4	4.3	5.0	5.7	3.6	3.9
White blood cell x10^9^/L	8.4	2.21	4.02	2.77	3.03	3.7	3.7
Absolute neutrophil Count × 10^9^/L	6.82	1.1	1.40	0.36	*N* ^∗∗^	*N* ^∗∗^	^∗^
Platelets × 10^9^/L	144	133	210	33	227	137	66
Reticulocyte count	*R* ^∗∗∗^	^∗^	^∗^	0.7%	12.57%	^∗^	^∗^
Total bilirubin (direct) umol/L	*R* ^∗∗∗^ > indirect	48 (3.98)	33.59 (9.79)	6.4 (2.9)	78.18 (7.06)	30.5 (18.76)	80 > Indirect
LDH (60–252 U/L)	^∗^	2135	4057	57	2507	875	^∗^
Direct Coombs test	Negative	Negative	Negative	Negative	Negative	Negative	^∗^
Serum B_12_ levels (138–651 pmol/L)	*N* ^∗∗^	338	713	275	*N* ^∗∗^	*N* ^∗∗^	282
Serum folate levels (2.7–17 ng/mL)	*N* ^∗∗^	14.1	9.2	24.1	> 20	*N* ^∗∗^	10.5
Serum ferritin levels (21–274 ng/mL)	*N* ^∗∗^	113.9	57.56	^∗∗^	25.67	*N* ^∗∗^	^∗^
Urinalysis	3 + blood −ve urobilinogen −ve bilirubin	^∗^	3 + blood 1 + urobilinogen −ve bilirubin	^∗^	3 + blood −ve urobilinogen −ve bilirubin	^∗^	2 + blood −ve urobilinogen −ve bilirubin

^∗^Results not available.

^∗∗^ *N* documented normal but result figure not in file.

^∗∗∗^ *R* documented raised but actual figure not available.

All patients had a bone marrow aspirate which was mostly reported as hypercellular, with few noted to have dyserythropoiesis. Two patients (Patients 1 and 2), who underwent repeat bone marrow examinations at different time points, demonstrated dynamic changes, with initial hypocellular marrow later evolving to hypercellularity. Patient 4 presented with pancytopenia, and bone marrow aspirate and trephine biopsy showed marked hypocellularity (< 5%) without increased blasts, consistent with very severe aplastic anaemia (VSAA) based on Camitta criteria. Molecular testing did not identify mutations associated with inherited bone marrow failure syndromes. Patient 7 demonstrated reduced megakaryopoiesis with erythroid hyperplasia, raising concern for an inherited bone marrow failure syndrome; however, molecular testing could not be performed due to financial constraints.

The diagnosis of PNH was confirmed by flow cytometric analysis in all seven patients. Six patients demonstrated large PNH clones, with more than 90% of neutrophils and monocytes lacking GPI‐anchored proteins (Table 3). As expected, the proportion of affected red blood cells varied widely (18.3%–90.1%), likely reflecting differences in timing of testing relative to haemolytic episodes and recent blood transfusions.

**TABLE 3:** FLAER flow cytometry.

Patient 1	99.3% neutrophils lack FLAER CD24, CD55 and CD 59
	99.3% monocytes lack FLAER and CD14
	90.1% red cells lack CD 59.

Patient 2	99% population of neutrophils lacked FLAER and CD24,
	98.8% monocytes lacked FLAER and CD 14
	26% red cells lacked expression of CD59.

Patient 3	99.4% neutrophils lack FLAER CD24, CD55 and CD 59
	99.4% monocytes lack FLAER and CD14
	18.3% red cells lack CD 59.

Patient 4	Large PNH clone > 90%
	NB: detailed report not available

Patient 5	96% neutrophils lacked expression of FLAER, CD 24 and CD16.
	94% monocytes lacked expression of CD 14 and FLAER
	32% red cells lacked CD59.

Patient 6	87% neutrophils lacked expression of FLAER and CD 24.
	87% monocytes lacked expression of CD 14 and FLAER
	^∗^Done at different lab, RBC not included.

Patient 7	99.3% neutrophils lack FLAER, CD24 and CD157
	99.7% monocytes lack FLAER, CD14 and CD157
	28.8% red cells lack CD 59.

^∗^The note indicates why the rec cell % is missing as the PNH flow cytometry was run in a different lab from other cases.

In addition, a 52‐year old female patient presented with seizures secondary to cerebral venous sinus thrombosis and haemolytic anaemia. Direct Coombs testing and autoimmune screening were negative, with normal serum folate and vitamin B12 levels. The bone marrow examination revealed a hypercellular marrow without abnormal infiltration. However, confirmatory FLAER‐based flow cytometry could not be performed due to financial constraints, and this patient was therefore not included in the confirmed PNH cohort in our case series. The case is mentioned for illustrative purposes, highlighting the diagnostic challenges encountered when patients have clinical features suspicious for PNH but confirmatory testing cannot be performed in resource‐limited settings.

Management strategies varied according to disease severity and associated cytopenias. Most patients were managed with supportive care mainly red cell transfusions for symptomatic anaemia and folic acid supplementation. Patient 4, diagnosed with VSAA, was treated with EPAG and cyclosporine, resulting in transfusion independence and improved blood counts. He subsequently underwent a matched sibling donor allogeneic stem cell transplant outside the country and remains well posttransplant. Patient 7, who also had significant cytopenias, was initiated on EPAG and cyclosporine and continues to be monitored for treatment response.

## 3. Discussion

Our study findings were notable for a younger patient cohort with a median age of 23 years (range 17–38 years) and a male predominance. This contrasts with data from the international PNH registry, which reported a median age of 42 years, while most published studies describe an age range between 30 and 50 years with a slight female predominance [2, 6, 16–19]. Although our cohort demonstrated a male predominance, this observation should be interpreted cautiously given the small sample size and may not reflect the true sex distribution of PNH in the wider population. All patients were of African descent. While regional differences in PNH presentation have been reported such as older age at presentation and greater marrow aplasia among Asian populations, the contribution of ethnicity to disease phenotype remains incompletely understood [16].

There was a delay in the diagnosis of PNH in our cohort with a mean duration of 5.02 years (range 2 months to 10 years) from initial clinical presentation to diagnosis. This finding is consistent with observations reported in other African studies [9, 10, 13]. Previous reports have attributed delayed diagnosis to the heterogeneous clinical presentation of PNH, its rarity, an initial focus on symptomatic management rather than definitive investigation and limited access to specialised diagnostic resources [9, 10, 13]. Similar challenges were encountered in our cohort.

The clinical presentation of PNH is heterogeneous and often nonspecific despite the classic triad of intravascular haemolysis, thrombosis and bone marrow failure. Disease course can range from indolent symptoms over many years to aggressive disease with life‐threatening complications [16]. Prior studies have demonstrated a correlation between clinical phenotype and clone size, with larger PNH clones more commonly associated with classical haemolytic diseases [5, 20, 21]. Consistent with this, all patients in our series had large PNH clones, with most exceeding 95% in granulocytes and monocytes. Symptomatic anaemia was universal and often severe, with haemoglobin levels as low as 2.4 g/dL, reflecting a combination of intravascular haemolysis and, in some cases, bone marrow failure [2].

Haemolysis in PNH leads to free haemoglobin release, overwhelming haptoglobin binding capacity and resulting in nitric oxide depletion. This results in smooth muscle dysfunction which may present as dysphagia, oesophageal spasms, abdominal pain and erectile dysfunction [3]. Abdominal pain was reported by two patients. Dark urine due to haemoglobinuria, particularly noted at night, was reported by the majority of patients and remains a key clinical clue, especially in resource‐limited settings [2]. Thrombosis, a major cause of morbidity and mortality in PNH, occurs in up to 40% of patients and predominantly affects the venous system, often at unusual sites such as the hepatic, portal, mesenteric and cerebral veins [2, 22]. One patient in our cohort presented with mesenteric vein thrombosis leading to bowel ischaemia, underscoring the severe consequences of thromboembolic disease. The increased thrombotic risk associated with large PNH clones is multifactorial and includes complement‐mediated platelet activation, nitric oxide depletion, endothelial dysfunction, impaired fibrinolysis and proinflammatory cytokine release [2, 22].

Evaluation of suspected PNH in our cohort demonstrated laboratory evidence of intravascular haemolysis, with haemoglobinuria with minimal bilirubin or urobilinogen serving as an important clue. Bone marrow evaluation aided in exclusion of other haematologic disorders, particularly in patients with cytopenias. The definitive diagnosis of PNH required flow cytometry demonstrating deficiency of GPI‐anchored proteins in at least two haematopoietic cell lineages [3]. While older assays such as the Ham test are now largely obsolete due to limited sensitivity, FLAER‐based flow cytometry is highly sensitive and specific for CD55 and CD59 on erythrocytes, monocytes and granulocytes. Although all cases were ultimately confirmed by flow cytometry, financial constraints contributed to delays in diagnosis.

Management of PNH has evolved from supportive care to targeted therapies such as complement inhibition. C5 inhibitors such as eculizumab and ravulizumab reduce intravascular haemolysis, transfusion requirements and thrombotic risk leading to improved survival [2, 18, 23–27]. However, these therapies require lifelong administration, do not address bone marrow failure and increase susceptibility to meningococcal infection, necessitating vaccination prior to treatment [28]. Newer agents targeting proximal complement pathways, including pegcetacoplan and factor D inhibitors, are emerging [29, 30]. These therapies remain largely inaccessible in low‐resource settings, as was the case for all patients in our series.

Consequently, management was primarily supportive, including transfusions, folate supplementation, cautious iron replacement and anticoagulation for thrombotic events [3]. Allogeneic stem cell transplantation remains the only curative therapy, particularly in patients with severe aplastic anaemia but carries significant morbidity and mortality [19]. In our series, one patient with VSAA underwent successful matched sibling transplantation and is now transfusion‐independent, while the remaining patients continue supportive care.

Historically, median survival for PNH was estimated at 15–20 years, with thrombosis as the leading cause of death [19]. Survival has improved substantially in the complement inhibitor era; however, outcomes remain poor for patients without access to these therapies [5, 31, 32]. Factors associated with the worse prognosis include older age, thrombosis, progression to pancytopenia and evolution to myelodysplastic syndrome or acute leukaemia [16, 31, 33].

## 4. Conclusion

Although sickle cell disease is the most common cause of haemolytic anaemia in Kenya, other causes of haemolysis should be considered; especially in patients with atypical features or poor response to standard management. Our findings highlight that PNH is present in Kenya, often affecting young patients and associated with prolonged diagnostic delay. Increased awareness, improved access to diagnostic testing and strategies to expand availability of definitive therapies are essential to improve outcomes in resource‐limited environments.

## Author Contributions

Matilda Ong’ondi: conceptualisation, summary of cases, literature review and write up and review and editing of write up. Otom Lavender: summary of one case and review and editing of the manuscript. Muraya Gathinji: summary of one case and editing of write up. Zaheer Bagha: summary of one case and review of write up.

## Funding

No funding was received for this review.

## Disclosure

All authors have read and approved the final version of the manuscript.

The corresponding author had full access to all of the data in this study and takes complete responsibility for the integrity of the data and accuracy of the data analysis.

## Ethics Statement

All patients gave consent for this publication, and the data were deidentified to ensure anonymity.

## Conflicts of Interest

The authors declare no conflicts of interest.

## Data Availability

The data that support the findings of this study are available from the corresponding author upon reasonable request.
